# Transient and resident *Salmonella*: A genomic approach to analyzing over a decade of sampling events from fish meal production and storage facilities

**DOI:** 10.1371/journal.pone.0327222

**Published:** 2025-07-01

**Authors:** Johnathan Likens, Jon W. Bell, Patricia Rabideau, Stephanie Haynes, Steven Wilson, Sandra Tallent, Eric W. Brown, Julie Haendiges

**Affiliations:** 1 National Seafood Inspection Laboratory, NOAA Fisheries, Pascagoula, Mississippi, United States of America; 2 Seafood Inspection Program, NOAA Fisheries, Silver Spring, Maryland, United States of America; 3 Division of Food Safety Genomics, United States Food and Drug Adminstration, Human Foods Program, College Park, Maryland, United States of America; Cornell University, UNITED STATES OF AMERICA

## Abstract

This study evaluates *Salmonella* contamination in feed mill production facilities over a 12-year period, analyzing collection events from 12 facilities predominantly located in the southeastern United States. The genomic data reveals a historical contamination rate, with 20% of collection events testing positive for *Salmonella*. Utilizing next generation sequencing this study evaluated the genetic diversity in the different facilities to determine whether the *Salmonella* serovars that were found are transient or resident. *Salmonella* serovars Montevideo, Ruiru, and Senftenberg were frequently detected, with Ruiru showing a particularly high predominance across multiple facilities, suggesting possible common sources of contamination including regional fishing waters and shared additives. The study also highlights the role of transportation and storage methods as a possible cause of cross-contamination. Future research should focus on identifying specific contamination sources and optimizing control measures to reduce *Salmonella* risks in fish meal production.

## Introduction

The presence of *Salmonella enterica* in feed milling facilities has been a longstanding concern. Research has demonstrated that *Salmonella* not only thrives but can persist for extended periods within these environments [1,2]. Some studies have even highlighted the phenomenon of certain *Salmonella* serovars becoming “mill adapted.” These adapted serovars employ various strategies, such as antimicrobial resistance, heat tolerance, and biofilm formation to enhance their survival in low-moisture conditions [3,4]. In addition to these adapted serovars, feed milling facilities often face other contamination risks. Common problems include poor equipment design, inadequate maintenance, insufficient sanitation practices, and inadequate pest control all of which contribute to contamination in low-moisture food production environments [5]. The National Seafood Inspection Laboratory (NSIL) has been the analytical monitoring laboratory for the National Oceanic & Atmospheric Administration (NOAA) Aquatic Animal By-Products Inspection Program (AABPIP) since 1968. NSIL performs various analyses on aquatic animal by-products, including a focus on detecting the presence of *Salmonella* in fish meal. The AABPIP is a component of NOAA’s Seafood Inspection Program (SIP) and participating facilities enrolled as Approved Establishments are inspected and sampled twice a year. Fish meal samples are taken from different stages of the manufacturing process, though most are collected from the final product during storage. NSIL maintains live cultures from all *Salmonella*-positive samples to verify contested results, creating a historical repository of bacterial contamination data spanning over a decade for each company tested.

In this study, Whole Genome Sequencing (WGS) was used to explore the genetic diversity within this extensive historical dataset. This approach enables the determination of whether the *Salmonella* isolates are due to transient contamination or have become established within the facilities and are considered resident strains. Differentiating between transient and resident strains is crucial for identifying potential long-term sources of contamination. Resident strains, once established, may persist within the production facilities, making them more challenging to eliminate and requiring more targeted interventions to prevent downstream contamination. These findings can inform targeted intervention strategies and improve contamination control measures in fish meal production facilities, ultimately enhancing product safety.

## Materials and methods

### Sample collection and isolate selection

NSIL reserves all culture-confirmed *Salmonella* isolates obtained from the routine sampling of AABPIP participating facilities. The majority of samples collected during routine monitoring originate from finished products held in storage. Occasionally, samples are also taken directly from the production line during active processing. The sampled commodities represent a range of products within the animal by-product sector, including fish meal, fish oil, bone meal, krill meal, and fish solubles. For this study, however, all analyzed samples were derived specifically from fish meal production facilities in final-stage fish meal products. These samples were collected following the USDC Seafood Inspection Manual Part 6 [6]. These isolate reserves were collected from 2010 − 2023. All *Salmonella* isolates were analyzed using a culture detection method and confirmed using a PCR confirmation method [7,8]. Following confirmation, the Tryptic Soy Broth (TSB) regrowth medium used during the verification process was utilized to prepare the isolate stock. A total of 1 mL of the confirmed isolate culture grown in TSB was mixed with 1 mL of a 50% glycerin solution in a sterile, 2 mL cryogenic tube. The resulting 1:1 mixture was then stored at ultra-low temperatures (−70°C to −80°C) to preserve the isolates for subsequent whole genome sequencing (WGS) analysis. For whole genome sequencing (WGS) selection, isolates were chosen to ensure representation from every facility sampled within the corresponding fiscal year. From each facility, a minimum of two isolates originating from the same collection event were included. This selection strategy yielded a total of 121 isolates from 12 distinct facilities. Of these, 112 met the analytical quality criteria for sequencing and were subsequently submitted to the NCBI under BioProject PRJNA939705.

Every culture-confirmed *Salmonella* isolate in the NSIL reserve library included metadata associated with each isolate (e.g., NSIL organism ID, Company sampled, Production lot, Collection date, Organism description). This metadata was collected at the time of sampling by the inspecting official in coordination with the company representative. Submission to NSIL was performed using a standardized sample submission form, and metadata was cross-verified against received samples upon arrival. Any discrepancies were resolved through direct confirmation with the collecting inspector prior to final acceptance. Once verified, metadata were linked to all downstream analytical results and archived digitally within NSIL’s secure, managed network.

### DNA extraction and whole-genome sequencing

Genomic DNA was obtained by aseptically scraping frozen isolate reserves onto brilliant green agar (BD, Franklin Lakes, NJ, USA) and incubated at 35°C overnight. A single colony was removed and inoculated in 10 mL TSB and incubated at 35°C overnight. DNA was extracted using the Qiagen QIAcube Connect and the DNeasy® Blood & Tissue kit (Qiagen, Germantown, MD, USA) following the bacterial pellet protocol. DNA concentrations were determined using the Qubit 4 fluorometer and the Double Stranded DNA High Sensitivity (dsDNA HS) assay kit (Thermo Fisher Scientific, Waltham, MA, USA). Library preparation was completed following the manufacturing guidelines for the Illumina® DNA Prep and the IDT® for Illumina® DNA/RNA UD Indexes (Illumina, San Diego, CA, USA). Paired-end sequencing (2 x 300 bp) was completed using the MiSeq Reagent kit v3 (600-cycle) on the Illumina® MiSeq.

### Data analysis and quality control

Raw FASTQ files from the forward and reverse reads were uploaded to the Galaxytrakr platform (https://galaxytrakr.org/). The raw data quality was assessed using FastQC v0.11.9 (https://www.bioinformatics.babraham.ac.uk/projects/fastqc/), assembled with SPAdes v3.12.0, and characterized using MLST v2.22 (https://github.com/tseemann/mlst) and SeqSero2 v1.2 [9,10]. The output assemblies from SPAdes were quality checked using Quast v5.2.0 [11]. The data quality was assessed by the following metrics: genome coverage, average Q score, assembly contig numbers, and genomic length of assemblies. Data that did not meet sequencing criteria of> 30x genome coverage, and/or average Q score for R1 or R2 ≥ 30 were excluded. In addition, any assemblies that had > 300 contigs, or incorrect genomic length (> 5.3 Mbp) and were not confirmed to be *Salmonella sp*. by MLST or SeqSero were not further analyzed. These quality control guidelines were defined based on the quality control assessment for microbial genomes: Galaxytrakr workflow (https://www.protocols.io/view/quality-control-assessment-for-microbial-genomes-g-5jyl8mj16g2w/v4).

The sequence data was submitted to the National Center for Biotechnology Information (NCBI) under BioProject PRJNA939705. The data was viewed in the NCBI Pathogen Detection Browser (https://www.ncbi.nlm.nih.gov/pathogens/) and the phylogenetic trees for a selection of isolates of interest were exported. Isolates of interest were selected based on a combination of parameters, including serovar designation, single nucleotide polymorphism (SNP) distance, geographic region of the facility, and associated parent company. These criteria enabled the construction of a plausible timeline of contamination events across the selected facilities. Antimicrobial resistance (AMR) genes and stress tolerance genes were computed by NCBI using AMRFinderPlus [12]. The dataset table was exported from NCBI Pathogen Detection browsers with these results.

### Resident vs. transient determination

Isolates were categorized into two different contamination event categories for comparisons: resident and transient. Resident serovars were categorized as serovars that were collected at least 90 days apart from the same facility and ≤ 50 Single Nucleotide Polymorphisms (SNPs) of one another [13,14]. Transient serovars were categorized as serovars that were from collection events < 89 days apart from the same facility and had a SNP difference of greater than > 50 SNPs.

## Results

### Fish meal facility sampling

**Fig 1:**
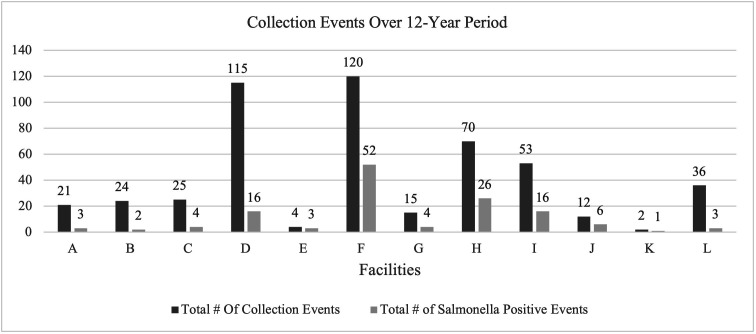
Total number of collection events and positive events for each facility. A positive event is defined as an event yielding a positive *Salmonella* isolate.

Sampling histories from 12 different facilities were examined over a 12-year period, focusing on the frequency a positive *Salmonella* isolate was found during a collection event. (Fig 1). After calculating the total number of facilities for each fiscal year, facilities were identified that were managed by larger parent companies. The 12 fish meal facilities belonged to six different parent companies. Each facility is inspected and sampled separately, regardless of whether they are managed by a larger parent company. The largest parent company oversees five different facilities in two different regions (Midwest and Southeast). The fish meal facilities were located in the Southeast (50%), Northwest (42%), and Midwest (8%). These facilities were primarily production facilities (91%) for aquatic animal by-products, while the others are solely storage facilities (9%). All of the facilities were sampled at least twice annually while participating in the AABPIP program except for the non-production storage facilities which were only required to be sampled once. Among the facilities that reported at least 20 collection events in the dataset, an average of 20% of these events yielded a *Salmonella* isolate. Additional audits were required for facilities that export fish meal product out of the U.S. These additional audits result in a greater number of collection events for some of the facilities. No direct correlation was found between the number of collection events and the number of positive samples in this dataset.

The samples collected were from various steps in the aquatic animal by-product production process (e.g., final product storage, on-line product, process equipment, and transportation equipment), A majority of isolates were sourced from final product storage or on-line product samples (88%). All of the samples analyzed were derived from fish meal samples, composed of fish species Menhaden, Pollock, Haddock, and Whiting. There were three facilities with a single collection event due to operations ceasing or a change in ownership over the sample collection period.

### WGS of *Salmonella* isolates

**Fig 2:**
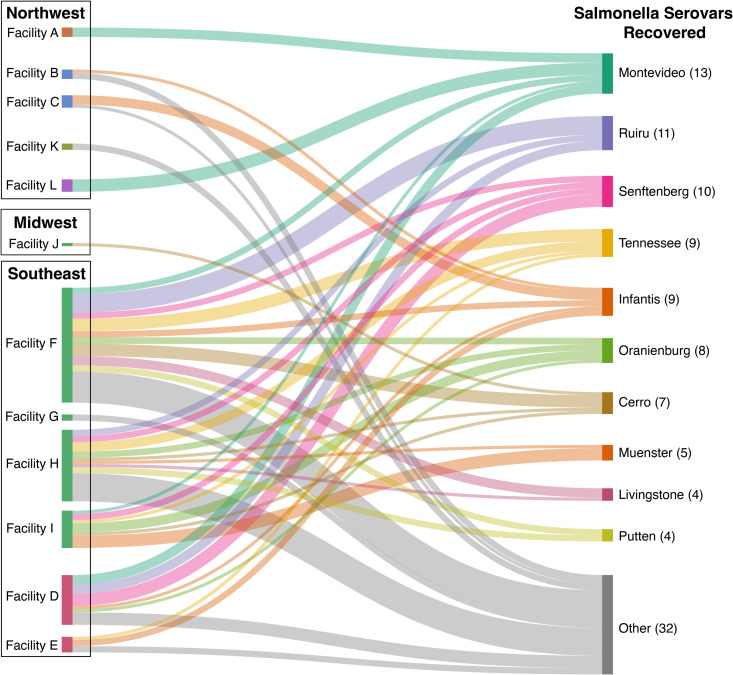
A Sankey plot showing the different servoars that were identified from the WGS of *Salmonella* isolates recovered from the different facilities. Facilities with the same color node belong to the same parent company (Parent Company 1: Facility B and C; Parent Company 2: Facility F, G, H, I, and J; and Parent Company 3: Facility D and E.

A total of 121 isolates were sequenced, while only 112 isolates passed analytical quality standards and were uploaded to NCBI under the BioProject PRJNA939705 (S1 Table). The majority of the sequenced isolates were sourced from the Southeast region (84.8%), followed by the Northwest region (14.3%), and lastly the Midwest region of the U.S. (0.9%). The nine isolates that failed quality parameters were due to non-*Salmonella* species identification or due to a mixed culture of bacterial species. The two non-*Salmonella* species found were *Citrobacter freundii* and *Enterobactor cloacae.* The majority of the isolates were *Salmonella enterica* subsp*. enterica* (97.3%) and included 27 different serovars. Fig 2 illustrates the serovars identified from the different facilities and their geographical region. Three isolates were typed as *Salmonella enterica* subsp*. arizonae* and were from the same parent company but different facilities and collection events. The most common *Salmonella enterica* subsp*. enterica* serovars were Montevideo (11.6%), Ruiru (9.8%), and Senftenberg (8.9%).

**Fig 3:**
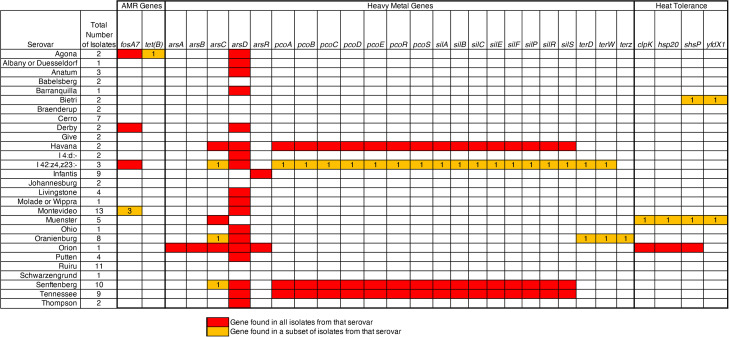
Presence/Absence data for AMR and stress genes identified by AMRFinderPlus in the 112 *Salmonella* isolates sequenced in this study. Red boxes denote the gene was found in 100% of the isolates from that serovar, while the orange boxes are for a subset. For the orange boxes, the number of isoaltes that were identified is listed.

AMRfinder Plus identified the following AMR and stress genes in all 112 isolates: *mdsA* (AMR), *mdsB* (AMR), *asr* (stress), *golS* (stress), and *golT* (stress), Fig 3 provides a summary of all other identifiedresistance genes. Ten isolates contained genes that conferred Fosfomycin resistance, and one isolate showed the prescence of Fosfomycin and tetracycline resistant genes. Arsenic resistance genes were found in 83 (74%) of the isolates, and 22 (19.6%) isolates contained the Copper Homeostasis and Silver Resistance Island (CHASRI) [15] genes. Arsenic levels in fish and seafood are often higher than those typically found in the surrounding environment. Arsenic has been previously identified in *Salmonella* Bareilly which was associated with bacterial contaminated tuna [16]. The presence of arsenic resistance genes in these isolates may link the *Salmonella* isolates to a marine origin. Genes associated with the Locus of Heat Resistance (*hsp20, clpK, yfdX1* and *shsP*) were also found in three isolates from Facility F and H, which may be a key factor in their persistence in this environment [17]. Fish meal is a heat dried, low moisture commodity product, and previous studies have shown that desiccated *Salmonella* has a higher tolerance to thermal treatment [18]. Preventive controls, such as thermal processing, to reduce microbial load in fish meal may require further review at these facilities to ensure adequate reduction of microbial load in the final product.

### Facility resident vs. transient contamination events

The majority of the *Salmonella* serovars (61%) were found in multiple facilities while only 39% occurred in a single facility. An average of five different *Salmonella* serovars were found in all 12 fish meal facilities. An average of three serovars were characterized as transient contamination events and two serovars were characterized as resident strains in these facilities. A majority of the facilities (58%) were found to have resident strains of *Salmonella*. Additionally, a majority of the facilities that were characterized as harboring a resident strain (57%) contained multiple resident serovars in the same facility (Table 1). All classified resident serovars were found to be within a maximum of 25 SNPs difference between each other. The three most prevalent serovars Montevideo, Ruiru, and Senftenberg provided the clearest examples of long-term harborage across various facilities.

**Table 1:** Resident serovars identified in fish meal facilities.

Facility	Resident Serovar	Collection Year	Sample ID	NCBI SNP Distance
**A**	Montevideo	2014	NSIL140588−1	0–13 SNPs
		2014	NSIL150651−1	
		2015	NSIL150732−1	
**C**	Infantis	2014	NSIL140538−1	5–15 SNPs
		2021	NSIL221956−1	
		2023	NSIL232078−1	
**D**	Anatum	2020	NSIL211853−1	2 SNPs
		2021	NSIL211910−1	
	Montevideo	2018	NSIL181358−1	1–14 SNPs
		2018	NSIL181358−2	
		2021	NSIL211897−1	
	Ruiru	2010	NSIL100011−1	2–16 SNPs
		2010	NSIL100011−2	
		2018	NSIL191464−2	
	Senftenberg	2018	NSIL191464−1	0–14 SNPs
		2019	NSIL191579−1	
		2019	NSIL191579−2	
		2019	NSIL201724−2	
**F**	Braenderup	2014	NSIL150637−1	3 SNPs
		2015	NSIL160788−1	
	I 42:z4,z23:-	2018	NSIL191439−2	10 SNPs
		2022	NSIL222050−1	
	Infantis	2017	NSIL171112−1	2 SNPs
		2020	NSIL201763−2	
	Livingstone	2014	NSIL140518−1	15 SNPs
		2015	NSIL160788−2	
		2018	NSIL191454−1	
	Putten	2014	NSIL140518−2	2 SNPs
		2014	NSIL150637−2	
	Ruiru	2010	NSIL100019−2	0–20 SNPS
		2015	NSIL150736−1	
		2015	NSIL150736−2	
		2016	NSIL160889−1	
		2017	NSIL171112−2	
		2018	NSIL181343−1	
	Tennessee	2018	NSIL181356−1	3 SNPs
		2019	NSIL201704−1	
**H**	Derby	2018	NSIL181340−1	2 SNPs
		2018	NSIL191434−1	
	Putten	2018	NSIL181353−1	7 SNPs
		2019	NSIL191593−1	
	Senftenberg	2017	NSIL171136−1	5 SNPs
		2018	NSIL191434−3	
	Tennessee	2014	NSIL140581−2	0–19 SNPs
		2016	NSIL160929−1	
		2016	NSIL160929−2	
**I**	Oranienburg	2016	NSIL160913−1	3 SNPs
		2018	NSIL181336−2	
	Senftenberg	2010	NSIL100026−2	8 SNPs
		2020	NSIL201826−1	
**L**	Montevideo	2017	NSIL171157−1	1–2 SNPs
		2018	NSIL181407−1	
		2018	NSIL181407−2	
		2019	NSIL191530−1	

### *Salmonella* Ruiru

**Fig 4:**
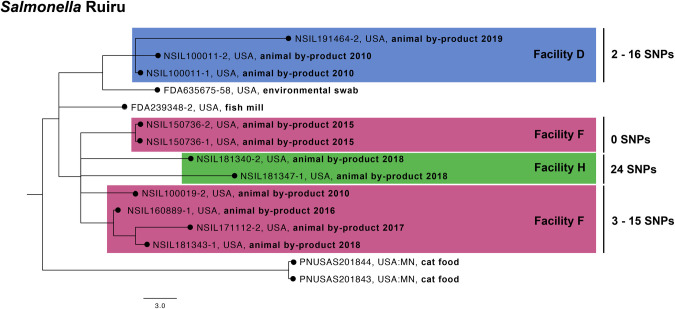
Phylogenetic tree of with NCBI SNP cluster PDS000031989 containing *Salmonella* Ruiru isolates from Facilities D, F, and H. A newick file was exported of the selected isolates from the NCBI Pathogen Detection Browser. SNP distances was determined in NCBI Pathogen Detection.

The NCBI SNP cluster PDS000031989 contains isolates collected from Facility D, F, and H (Fig 4). *S.* Ruiru was initally identified in the dataset on January 21, 2010 in Facility D (NSIL100011–1 and NSIL100011–2, as shown in Fig 4). *S.* Ruiru remained undetected in Facility D for 8 years until December 5, 2018. The SNP difference among the *S.* Ruiru isolates from Facility D averaged 11 SNPs. On January 27, 2010, *S.* Ruiru was also detected in Facility F (NSIL100019–2, as shown in Fig 4). In Facility F, this serovar was consistently detected on an annual basis from 2015 to 2018. The SNP difference among the *S.* Ruiru isolates from Facility F also averaged 11 SNPs, similar to Facility D. On July 10, 2018 (NSIL181340–2), *S.* Ruiru was initally detected in Facility H, and this serovarwas identified twice during that year. The SNP difference among the *S.* Ruiru isolates from Facility H averaged 24 SNPs. This data suggests that a possible bacterial contamination event occurred in early 2010 affecting both Facility D and Facility F, which are managed by different parent companies. The same parent company operates Facilities F and H, and *S.* Ruiru was only detected annually at Facility F (2015–2018) until it was identified at both facilities in 2018.

This SNP cluster also contained isolates collected by the FDA (FDA635675−58 and FDA239348−2). FDA635675−58 was isolated from an environmental swab in 2010 from Firm A, a pet food manufacturer. This isolate differs from the fish meal Facility D isolates by 8–10 SNPs. FDA239348−2 was isolated from fish mill Firm B in 2003, and is 13–15 SNPs away from the isolates from Facility D.

The origin of the initial bacterial contamination event at both Facilities D and F is unclear, and complicates efforts to determine why this serovar has persisted in these facilities. However, this data indicates that *S.* Ruiru can thrive in low moisture environments and endure for extended periods. The genetic similarity among *S.* Ruiru isolates from Facilities D, H, and F suggests a common source of the *Salmonella* isolates, supporting the hypothesis that regional fishing waters, additives, or transportation/distribution could be responsible for the widespread presence of this serovar across the facilities. Another indication of a common source for the *S.* Ruiru serovar is the geographical locations of Facilities D, H, and F. All of these facilities are located within ~200 miles of each other in the Southeastern United States. Shared harvesting grounds and product distribution channels may be another indication as to why such a dense clustering of this particular serovar was found across multiple facilities.

### *Salmonella* Senftenberg

**Fig 5:**
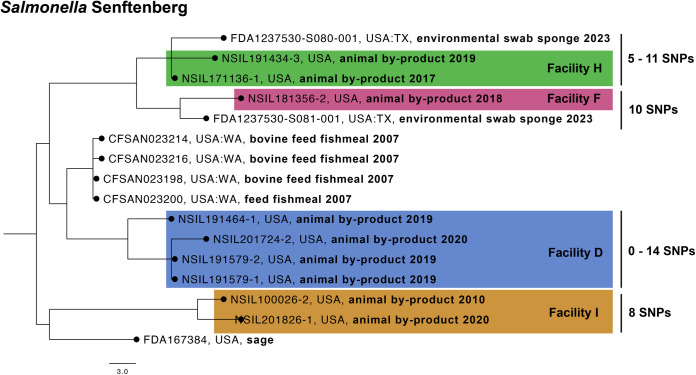
Phylogenetic tree of with NCBI SNP cluster PDS000032100 containing *Salmonella* Senftenberg isolates from Facilities D, F, H, and I. A newick file was exported of the selected isolates from the NCBI Pathogen Detection Browser. SNP distances was determined in NCBI Pathogen Detection.

The NCBI SNP cluster PDS000032100 contains isolates collected from fish meal Facilities D, F, H, and I (Fig 5). *S.* Senftenberg was initally identified in this dataset on February 18, 2010, in Facility I (NSIL100026−2), and was not detected again until September 24, 2020 (NSIL201826−1). The SNP difference among the *S*. Senftenberg isolates from Facility I averaged eight SNPs. *S.* Senftenberg was next detected on August 30, 2017 in Facility H (NSIL171136−1), and remained undetected until October 16, 2018 (NSIL191434−3). The SNP difference among the *S*. Senftenberg isolates from Facility H averaged five SNPs. On July 20, 2018, *S.* Senftenberg was detected for the only time in Facility F (NSIL18356−2). On December 5, 2018, *S.* Senftenberg was initally detected in Facility D (NSIL191464−1) and would be detected twice more in June 03, 2019 (NSIL191579−1; NSIL191579−2) and November 20, 2019 (NSIL201724−2). The SNP difference among the *S.* Senftenberg isolates from Facility D averaged 7 SNPs. This SNP cluster also contained two isolates that were collected by the FDA (FDA1237530-S080-001 and FDA1237530-S081-001) from Firm C, a pet food manufacturer. These *S.* Senftenberg isolates were collected in 2023 and differ from the NSIL fish meal isolates by 10–11 SNPs.

The data from the *S.* Senftenberg isolates suggests that a potential bacterial contamination event occured in early 2010 affecting Facility I. Subsequently, isolates from Facilities H and F, which are operated by the same parent company, indicated the presence of a *S.* Senftenberg strain highly related to the one found in Facility I. In late 2018 at Facility D, managed by a different parent company, this highly related *S.* Senftenberg strain was also detected. Shared fishing grounds is an unlikely contributor to the strain similarity due to the different locations of these facilities. Facility I is located ~1000 miles from Facility H, F, and D on the Eastern United States. Facility I shares the same parent company as Facility H and F, and the final fishmeal products may have been distributed between all three locations. The source of the initial contamination remains unclear, which complicates efforts to determine why this serovar has persisted in these facilities.

### *Salmonella* Montevideo

**Fig 6:**
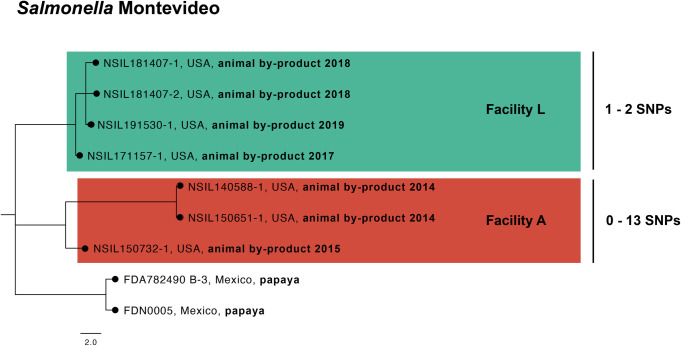
Phylogenetic subtree of with NCBI SNP cluster PDS000172527 containing *Salmonella* Montevideo isolates from Facilities A and L. A newick file was exported of the selected isolates from the NCBI Pathogen Detection Browser. SNP distances was determined in NCBI Pathogen Detection.

The NCBI SNP cluster PDS000172527 contains isolates collected from Facility A and L (Fig 6). *S.* Montevideo was initally detected in our dataset on August 02, 2014, in Facility A (NSIL140588−1), and would later be detected two more times on October 30, 2014 (NSIL150651−1) and June 24, 2015 (NSIL150732−1). The SNP difference among the *S.* Montevideo isolates for Facility A averaged 9 SNPs. The next detection of this serovar was on September 14, 2017, in Facility L (NSIL171157−1, as shown in Fig 6), and was detected twice more on September 14, 2018 (NSIL181407−1; NSIL181407−2) and March 26, 2019 (NSIL191530−1). The SNP difference among these isolates for Facility L averaged 2 SNPs.

This data from the *S.* Montevideo isolates suggests that a possible bacterial contamination event occurred in 2014 affecting Facility L, and another event followed in 2017 affecting Facility A. These facilities are not operated by the same parent company but are located within a few miles of each other in the Northwestern U.S. The genetic similarity among *S.* Montevideo isolates from Facilities L and A suggests a common source, and supports the hypothesis that regional fishing waters, additives, or transportation/distribution are potentially responsible for the widespread presence of this serovar across the facilities.

## Discussion

Over the 12-year period, 497 sample collection events were analyzed from 12 different facilities. Twenty percent of these events tested positive for *Salmonella* bacteria, highlighting an ongoing challenge with *Salmonella* contamination in fish meal production facilities. Almost 84% of these facilities are located in the southeastern U.S. These facilities generally store bulk final products in warehouses after fish meal processing, and these products may be vulnerable to external factors such as high humidity and pest contamination. In contrast, facilities with lower *Salmonella* positivity rates in the northwestern and southwestern U.S, usually store final products in sealed bags. This final product packaging and storage may influence or reduce the risk of *Salmonella* contamination. The varying storage methods could also influence the contamination risk during the handling and distribution of final products. Additionally, the practice of comingling final products by facilities managed by the same parent company is common. The goal of this practice is to reduce costs by consolidating bulk shipments. This comingling may explain the detection of the same *Salmonella* serovars across multiple facilities within the same company. Our findings are similar to recent studies in marine environments showing that resistance genes can persist and spread among bacteria. For example, *Escherichia coli* isolated from marine systems were found to carry both arsenic and antibiotic resistance genes on the same plasmids, suggesting that these bacteria can act as reservoirs for resistance traits. Other research has shown that some eukaryotic arsenic resistance genes likely originated from marine bacteria through horizontal gene transfer [19,20]. These examples highlight the role of mobile genetic elements in enabling bacteria to acquire resistance traits and persist over time in both environmental and food production settings.

Several hypotheses exist to explain the occurrence of shared isolates among different parent companies. Harvesting fish from common fishing grounds could result in multiple parent companies encountering the same source of *Salmonella*. The detection of shared isolates would indicate that these multiple facilities harbor the same serovar for an extended period of time. These multiple parent companies might also be using the same antioxidant additive, which is typically bought in bulk and used across all facilities within each parent company. Another possible explanation could be the involvement of third-party companies in the transportation or distribution of final fish meal products across multiple facilities. These parent companies often rely on contracted third parties for both domestic and international transport of their products.

Shared fishing waters could be a potential source of contamination for these facilities, particularly those processing aquatic animal by-products from Gulf menhaden. During the Gulf menhaden harvest season, which takes place in northern Gulf of Mexico waters, fishing activities are concentrated in a nearshore environment. This setting could expose the fishing vessels to *Salmonella* spp., which could introduce these bacteria into the fish meal products. Additional research is necessary for facilities producing aquatic animal by-products from pollock, haddock, and whiting to determine if shared waters could be a possible source of shared contamination. This research should investigate whether the harvest waters and fish species in these areas present exposure risks comparable to those associated with Gulf mendaden in the northern Gulf of Mexico.

The use of antioxidant additives may also introduce bacterial contamination in final fish meal products. Fish meal, which is rich in omega-3 fatty acids, is particularly susceptible to lipid oxidation and, in extreme cases, spontaneous combustion. These additives prevent oxidation, stabilize the fish meal, and maintain the nutrient quality of the final fish meal product. Antioxidants such as ethoxyquin are frequently used for this purpose. Additionally, various oils, such as canola and rosemary oil, may be incorporated into fish meal to reduce oxidation and enhance nutritional value. However, the addition of these additives may introduce bacterial contaminants, either through the additives themselves or from the equipment used to apply them.

The transportation of final aquatic animal by-products introduces another risk of potential contamination. After production is completed, fish meal is generally loaded onto rail cars, trucks, and barges for delivery. Production companies often rely on third-party transportation including the cleanliness and sterility of the conveyances provided. The transportation companies are required, and depended on, to clean and sterilize the conveyances between shipments. These transportation companies may need to improve and/or update cleaning protocols, enhance equipment maintenance, and implement spot testing of transportation lines to reduce contamination risks and verify that their quality control efforts are adequate. These actions could help to minimize contamination introduced into these production facilities. Multiple production facilities may also use the same transportation providers, potentially complicating contamination control efforts.

This evaluation and analysis demonstrates the continued challenges of *Salmonella* contamination confronting fish meal production and storage facilities. Seven of the 12 facilities studied have been shown to retain a resident *Salmonella* serovar for greater than 90 days, and most consistently do so for many years. The top three serovars found in this study were Montevideo (11.6%), Ruiru (9.8%) and Senftenberg (8.9%), and showcase a common theme throughout the dataset. These serovars were more prevalent in this study’s dataset than they were in the *Salmonella* database on the NCBI Pathogen Detection Browser. *S.* Ruiru comprises only 0.01% of the total number of *Salmonella* isolates in the NCBI Pathogen Detection database (as of September 2024). Some of the isolates from this study are highly related to previous sequenced isolates from manufactured pet food products. Highly related serovars of *S.* Senftenberg (0.6% of the NCBI Pathogen Detection *Salmonella*) from environmental swab sponges, bovine feed fish meal, and feed fish meal from Texas and Washington were also detected in this study’s fish meal isolates. These relationships highlight the importance of a One Health focus on pathogen control and the occuerence of ingredient cross-contamination from one facility to another. *Salmonella* serovars Montevideo and Senftenberg have previously been associated with persistence in low-moisture foods [21,22]. *Salmonella* bacteria in a desiccated state have been shown to develop increased thermal tolerance [18]. The presence of these serovars in the samples from fish meal indicates that further research is needed to determine potential persistence factors and provide insights into enhanced survivability in final products.

## Conclusion

This comprehensive, 12-year analysis of *Salmonella* contamination from 12 fish meal production and storage facilities highlights the challenges in managing *Salmonella* contamination risks in these facilities. The data highlights the ongoing risk of bacterial contamination in fish meal, and resulted in an average *Salmonella* detection rate of 20%. Higher rates were observed in facilities located in the southeastern U.S. This study reveals notable patterns of the presence of *Salmonella* serovars Montevideo, Ruiru, and Senftenberg in fish meal processing and storage facilities. Potential common sources of contamination, including shared regional fishing waters, antioxidant additives, and transportation networks, were identified. The similar SNP profiles of these *Salmonella* serovars across various facilities suggest that certain environmental or logistical factors impact the propagation of these strains. Future research should focus on investigating regional contamination sources to determine impacts of sanitation and maintenance protocols, additive usage, and transportation practices. Implementing targeted control measures and improving cross-facility coordination could significantly mitigate the risks associated with *Salmonella* contamination, and enhance product quality in fish meal production and storage facilities.

## Supporting information

S1 Table Metadata for the isolates sequenced in this study. ^1^All isolates contained the AMR genes mdsA and mdsB. ^2^All isolates contained the stress genes asr, golS and golT.
(PDF)
