# Supplementary material for: Transient and resident Salmonella: A genomic approach to analyzing over a decade of sampling events from fish meal production and storage facilities
Source: PLoS One. 2025 Jul 1;20(7):e0327222. doi: 10.1371/journal.pone.0327222 (PMC12212557; doi:10.1371/journal.pone.0327222)
Supplement: S1 Table — 1All isolates contained the AMR genes mdsA and mdsB. 2All isolates contained the stress genes asr, golS and golT. (PDF) [file pone.0327222.s001.pdf]

S1 Table: Metadata for the isolates sequenced in this study.

Strain ID	Region	Parent Company	Facility	Collection Year	AMR genotypes[1]	Stress genotypes[2]	SRA Accession	Computed Serotype
NSIL100011-1	SE	3	D	2010			SRR23941135	Ruiru
NSIL100011-2	SE	3	D	2010			SRR23941134	Ruiru
NSIL100018-1	SE	4	G	2010			SRR23941102	Johannesburg
NSIL100018-2	SE	4	G	2010			SRR23941091	Bietri
NSIL100019-2	SE	4	F	2010			SRR23941080	Ruiru
NSIL100026-1	SE	4	I	2010			SRR23941122	Schwarzengrund
NSIL100026-2	SE	4	I	2010		arsD,pcoA,pcoB,pcoC,pcoD,pcoE,pcoR,pcoS,silA,silB,silC,silE,silF,silP, silR,silS	SRR23941114	Senftenberg
NSIL140465-1	SE	4	F	2014		arsD	SRR23941113	Oranienburg
NSIL140465-2	SE	4	F	2014			SRR23941112	Cerro
NSIL140465-3	SE	4	F	2014			SRR23941111	Cerro
NSIL140469-1	SE	4	F	2014		arsD	SRR23941133	Oranienburg
NSIL140581-1	SE	4	H	2014			SRR23941132	Johannesburg
NSIL140581-2	SE	4	H	2014		arsD,pcoA,pcoB,pcoC,pcoD,pcoE,pcoR,pcoS,silA,silB,silC,silE,silF,silP, silR,silS	SRR23941110	Tennessee
NSIL140588-1	W	1	A	2014		arsD	SRR23941109	Montevideo

NSIL150635-1	SE	4	H	2014			SRR23941108	Cerro
NSIL150637-1	SE	4	F	2014			SRR23941107	Braenderup
NSIL150651-1	W	1	A	2014		arsD	SRR23941106	Montevideo
NSIL150665-1	W	2	B	2015		arsR	SRR23941105	Infantis
NSIL160866-1	SE	3	E	2016		arsD,pcoA,pcoB, pcoC,pcoD,pcoE, pcoR,pcoS,silA,sil B,silC,silE,silF,sil P, silR,silS	SRR23941104	Tennessee
NSIL160885-1	SE	3	D	2016		arsD	SRR23941103	Ohio
NSIL160885-2	SE	3	D	2016	fosA7.2	arsD	SRR23941101	Agona
NSIL160889-1	SE	4	F	2016			SRR23941100	Ruiru
NSIL160913-1	SE	4	I	2016		arsD	SRR23941099	Oranienburg
NSIL160929-1	SE	4	H	2016		arsD,pcoA,pcoB, pcoC,pcoD,pcoE, pcoR,pcoS,silA,sil B,silC,silE,silF,sil P, silR,silS	SRR23941098	Tennessee
NSIL160933-1	W	5	K	2016		arsD	SRR23941097	Thompson
NSIL160933-2	W	5	K	2016		arsD	SRR23941096	Thompson
NSIL171085-1	SE	3	E	2017		arsR	SRR23941095	Infantis
NSIL171085-2	SE	3	E	2017		arsR	SRR23941094	Infantis
NSIL171112-1	SE	4	F	2017		arsR	SRR23941093	Infantis

NSIL171136-1	SE	4	H	2017		arsD,pcoA,pcoB, pcoC,pcoD,pcoE, pcoR,pcoS,silA,sil B,silC,silE,silF,sil P, silR,silS	SRR23941092	Senftenberg
NSIL171157-1	W	6	L	2017		arsD	SRR23941090	Montevideo
NSIL181174-1	MW	4	J	2017			SRR23941089	Cerro
NSIL181312-1	SE	3	E	2018		arsD	SRR23941088	Molade or Wippra
NSIL181312-2	SE	3	E	2018		arsD	SRR23941087	Anatum
NSIL181336-1	SE	4	I	2018			SRR23941086	Cerro
NSIL181340-1	SE	4	H	2018	fosA7.3	arsD	SRR23941085	Derby
NSIL181343-1	SE	4	F	2018			SRR23941084	Ruiru
NSIL181358-1	SE	3	D	2018		arsD	SRR23941083	Montevideo
NSIL181407-1	W	6	L	2018		arsD	SRR23941082	Montevideo
NSIL181407-2	W	6	L	2018		arsD	SRR23941081	Montevideo
NSIL191434-1	SE	4	H	2018	fosA7.3	arsD	SRR23941079	Derby
NSIL191439-1	SE	4	F	2018		shsP,yfdX1	SRR23941131	Bietri
NSIL191444-1	SE	4	F	2018	fosA7	arsD	SRR23941130	Montevideo
NSIL191464-1	SE	3	D	2018		arsD,pcoA,pcoB, pcoC,pcoD,pcoE, pcoR,pcoS,silA,sil B,silC,silE,silF,sil P, silR,silS	SRR23941129	Senftenberg

NSIL191464-2	SE	3	D	2018			SRR23941128	Ruiru
NSIL191530-1	W	6	L	2019		arsD	SRR23941127	Montevideo
NSIL191621-1	W	2	B	2019			SRR23941126	Give
NSIL191621-2	W	2	B	2019			SRR23941125	Give
NSIL201812-1	SE	4	F	2020		arsD,pcoA,pcoB, pcoC,pcoD,pcoE, pcoR,pcoS,silA,sil B,silC,silE,silF,sil P, silR,silS	SRR23941124	Tennessee
NSIL201812-2	SE	4	F	2020		arsD,pcoA,pcoB, pcoC,pcoD,pcoE, pcoR,pcoS,silA,sil B,silC,silE,silF,sil P, silR,silS	SRR23941123	Tennessee
NSIL201826-1	SE	4	I	2020		arsD,pcoA,pcoB, pcoC,pcoD,pcoE, pcoR,pcoS,silA,sil B,silC,silE,silF,sil P, silR,silS	SRR23941121	Senftenberg
NSIL211833-1	SE	4	I	2020		arsD,pcoA,pcoB, pcoC,pcoD,pcoE,p coR,pcoS,silA,silB , silC,silE,silF,silP, silR,silS	SRR23941120	Tennessee
NSIL211897-1	SE	3	D	2021		arsD	SRR23941119	Montevideo
NSIL221956-1	W	2	C	2021		arsR	SRR23941118	Infantis

NSIL222010-1	W	2	C	2022			SRR23941117	Babelsberg
NSIL222050-1	SE	4	F	2022	fosA7	arsD	SRR23941116	I 42:z4,z23:-
NSIL222050-2	SE	4	F	2022		arsD	SRR23941115	Barranquilla
NSIL140465-4	SE	4	F	2014			SRR24190228	Cerro
NSIL140465-5	SE	4	F	2014			SRR24190227	Cerro
NSIL140518-1	SE	4	F	2014		arsD	SRR24190216	Livingstone
NSIL140518-2	SE	4	F	2014		arsD	SRR24190205	Putten
NSIL140538-1	W	2	C	2014		arsR	SRR24190194	Infantis
NSIL150637-2	SE	4	F	2014		arsD	SRR24190183	Putten
NSIL150732-1	W	1	A	2015		arsD	SRR24190177	Montevideo
NSIL150736-1	SE	4	F	2015			SRR24190176	Ruiru
NSIL150736-2	SE	4	F	2015			SRR24190175	Ruiru
NSIL160788-1	SE	4	F	2015			SRR24190174	Braenderup
NSIL160788-2	SE	4	F	2015		arsD	SRR24190226	Livingstone
NSIL160788-3	SE	4	F	2015	fosA7.2, tet(B)	arsD	SRR24190225	Agona
NSIL160929-2	SE	4	H	2016		arsD,pcoA,pcoB, pcoC,pcoD,pcoE, pcoR,pcoS,silA,silB,silC,silE,silF,silP, silR,silS	SRR24190224	Tennessee
NSIL171112-2	SE	4	F	2017			SRR24190223	Ruiru

NSIL181278-1	SE	4	H	2018		arsC,arsD,terD, terW,terZ	SRR24190222	Oranienburg
NSIL181278-2	SE	4	H	2018			SRR24190221	Babelsberg
NSIL181336-2	SE	4	I	2018		arsD	SRR24190220	Oranienburg
NSIL181340-2	SE	4	H	2018			SRR24190219	Ruiru
NSIL181341-1	SE	4	H	2018		arsC,clpK,hsp20, shsP, yfdX1	SRR24190218	Muenster
NSIL181341-2	SE	4	H	2018	fosA7	arsD	SRR24190217	I 42:z4,z23:-
NSIL181342-2	SE	4	H	2018		arsD	SRR24190215	I 4:d:-
NSIL181343-2	SE	4	F	2018		arsC,arsD,pcoA, pcoB,pcoC,pcoD, pcoE,pcoR,pcoS, silA,silB,silC,silE, silF,silP,silR,silS	SRR24190214	Havana
NSIL181347-1	SE	4	H	2018			SRR24190213	Ruiru
NSIL181348-1	SE	4	H	2018		arsD	SRR24190212	Livingstone
NSIL181353-1	SE	4	H	2018		arsD	SRR24190211	Putten
NSIL181356-1	SE	4	F	2018		arsD,pcoA,pcoB, pcoC,pcoD,pcoE, pcoR,pcoS,silA,sil B,silC,silE,silF,sil P, silR,silS	SRR24190210	Tennessee

NSIL181356-2	SE	4	F	2018		arsD,pcoA,pcoB, pcoC,pcoD,pcoE, pcoR,pcoS,silA,sil B,silC,silE,silF,sil P, silR,silS	SRR24190209	Senftenberg
NSIL181357-1	SE	4	H	2018		arsD	SRR24190208	I 4:d:-
NSIL181357-2	SE	4	H	2018		arsD	SRR24190207	Oranienburg
NSIL181358-2	SE	3	D	2018		arsD	SRR24190206	Montevideo
NSIL181366-1	SE	4	F	2018	fosA7	arsD	SRR24190204	Montevideo
NSIL181366-2	SE	4	F	2018		arsA,arsB,arsC,ars D, arsR,clpK,hsp20, shsP, yfdX1	SRR24190203	Orion
NSIL191434-2	SE	4	H	2018		arsC,arsD,pcoA, pcoB,pcoC,pcoD, pcoE,pcoR,pcoS, silA,silB,silC,silE, silF,silP,silR,silS	SRR24190202	Havana
NSIL191434-3	SE	4	H	2018		arsD,pcoA,pcoB, pcoC,pcoD,pcoE, pcoR,pcoS,silA,sil B,silC,silE,silF,sil P, silR,silS	SRR24190201	Senftenberg

NSIL191439-2	SE	4	F	2018	fosA7	arsC,arsD,pcoA, pcoB,pcoC,pcoD, pcoE,pcoR,pcoS, silA,silB,silC,silE, silF,silP,silR,silS, terD, terW,terZ	SRR24190200	I 42:z4,z23:-
NSIL191454-1	SE	4	F	2018		arsD	SRR24190199	Livingstone
NSIL191454-2	SE	4	F	2018		arsD	SRR24190198	Albany or Duesseldorf
NSIL191482-1	SE	4	I	2019		arsC	SRR24190197	Muenster
NSIL191482-2	SE	4	I	2019		arsC	SRR24190196	Muenster
NSIL191509-1	SE	4	I	2019		arsC	SRR24190195	Muenster
NSIL191509-2	SE	4	I	2019		arsC	SRR24190193	Muenster
NSIL191579-1	SE	3	D	2019		arsD,pcoA,pcoB, pcoC,pcoD,pcoE, pcoR,pcoS,silA,sil B,silC,silE,silF,sil P, silR,silS	SRR24190192	Senftenberg
NSIL191579-2	SE	3	D	2019		arsD,pcoA,pcoB, pcoC,pcoD,pcoE, pcoR,pcoS,silA,sil B,silC,silE,silF,sil P, silR,silS	SRR24190191	Senftenberg
NSIL191593-1	SE	4	H	2019		arsD	SRR24190190	Putten
NSIL191655-1	SE	3	D	2019		arsD	SRR24190189	Oranienburg

NSIL201704-1	SE	4	F	2019		arsD,pcoA,pcoB, pcoC,pcoD,pcoE, pcoR,pcoS,silA,sil B,silC,silE,silF,sil P, silR,silS	SRR24190188	Tennessee
NSIL201724-1	SE	3	D	2019		arsR	SRR24190187	Infantis
NSIL201724-2	SE	3	D	2019		arsD,pcoA,pcoB, pcoC,pcoD,pcoE, pcoR,pcoS,silA,sil B,silC,silE,silF,sil P, silR,silS	SRR24190186	Senftenberg
NSIL201763-1	SE	4	F	2020		arsC,arsD,pcoA, pcoB,pcoC,pcoD, pcoE,pcoR,pcoS, silA,silB,silC,silE, silF,silP,silR,silS	SRR24190185	Senftenberg
NSIL201763-2	SE	4	F	2020		arsR	SRR24190184	Infantis
NSIL211851-1	SE	4	I	2020	fosA7	arsD	SRR24190182	Montevideo
NSIL211851-2	SE	4	I	2020		arsD	SRR24190181	Oranienburg
NSIL211853-1	SE	3	D	2020		arsD	SRR24190180	Anatum
NSIL211910-1	SE	3	D	2021		arsD	SRR24190179	Anatum
NSIL232078-1	W	2	C	2023		arsR	SRR24190178	Infantis
